# Ocular Sequelae of Congenital Toxoplasmosis in Brazil Compared with Europe

**DOI:** 10.1371/journal.pntd.0000277

**Published:** 2008-08-13

**Authors:** Ruth E. Gilbert, Katherine Freeman, Eleonor G. Lago, Lilian M. G. Bahia-Oliveira, Hooi Kuan Tan, Martine Wallon, Wilma Buffolano, Miles R. Stanford, Eskild Petersen, for The European Multicentre Study on Congenital Toxoplasmosis (EMSCOT)

**Affiliations:** 1 Centre for Paediatric Epidemiology and Biostatistics, Institute of Child Health, London, United Kingdom; 2 Albert Einstein College of Medicine, Department of Epidemiology and Population Health, Bronx, New York, United States of America; 3 Congenital Infections, Department of Pediatrics, Pontificia Universidade Católica do Rio Grande do Sul, Porto Alegre, Brazil; 4 Laboratorio de Biologia do Reconhecer, Centro de Biociencias e Biotecnologicas, Universidade Estadual do Norte Fluminense, Campos dos Goytacazes, Goytacazes, Brazil; 5 Laboratoire de Parasitologies Exotique, Hôpital de la Croix Rousse, Lyon, France; 6 Perinatal Infection Unit, Dept of Pediatrics, University of Naples Federico II, Naples, Italy; 7 Department of Academic Ophthalmology, King's College London, London, United Kingdom; 8 Department of Infectious Diseases, Aarhus University Hospital, Aarhus, Denmark; CPqRR/Fiocruz, Brazil

## Abstract

**Background:**

Toxoplasmic retinochoroiditis appears to be more severe in Brazil, where it is a leading cause of blindness, than in Europe, but direct comparisons are lacking. Evidence is accumulating that more virulent genotypes of *Toxoplasma gondii* predominate in South America.

**Methods:**

We compared prospective cohorts of children with congenital toxoplasmosis identified by universal neonatal screening in Brazil and neonatal or prenatal screening in Europe between 1992 and 2003, using the same protocol in both continents.

**Results:**

Three hundred and eleven (311) children had congenital toxoplasmosis: 30 in Brazil and 281 in Europe, where 71 were identified by neonatal screening. Median follow up was 4.1 years in Europe and 3.7 years in Brazil. Relatively more children had retinochoroiditis during the first year in Brazil than in Europe (15/30; 50% versus 29/281; 10%) and the risk of lesions by 4 years of age was much higher: the hazard ratio for Brazil versus Europe was 5.36 (95%CI: 3.17, 9.08). Children in Brazil had larger lesions, which were more likely to be multiple and to affect the posterior pole (p<0.0001). In Brazil, visual impairment (<6/12 Snellen) was predicted for most affected eyes (87%, 27/31), but not in Europe (29%; 20/69, p<0.0001). The size of newly detected lesions decreased with age (p = 0.0007).

**Conclusions:**

*T. gondii* causes more severe ocular disease in congenitally infected children in Brazil compared with Europe. The marked differences in the frequency, size and multiplicity of retinochoroidal lesions may be due to infection with more virulent genotypes of the parasite that predominate in Brazil but are rarely found in Europe.

## Author Summary

*Toxoplasma gondii* is found throughout the world and is the most common parasitic infection in humans. Infection can cause inflammatory lesions at the back of the eye, which sometimes affect vision. These complications appear to be more common and more severe when people acquire infection in Brazil than in Europe or North America, but there have been no direct comparisons of patients identified and followed up in the same way. In this report, we compare children with congenital toxoplasmosis diagnosed at birth by universal screening in Europe and Brazil and followed up until the age of 4. We found that Brazilian children had a 5 times higher risk than European children of developing eye lesions and their lesions were larger, more numerous and more likely to affect the part of the area of the retina responsible for central vision. Two-thirds of Brazilian children infected with congenital toxoplasmosis had eye lesions by 4 years of age compared with 1 in 6 in Europe. These stark differences are likely to be due to the predominance of more virulent genotypes of the parasite in Brazil, which are rarely found in Europe.

## Introduction

Toxoplasmic retinochoroiditis appears to be more common in Brazil than in Europe or North America and more severe. It is a leading cause of blindness in Brazil[1] but not in Europe or North America.[2],[3] These differences are not adequately explained by high rates of postnatal or congenital infection in Brazil, as similar rates of infection have been observed in France and Eastern Europe.[4]–[8] Once infected, population-based studies of adolescents and adults, most of whom have postnatally acquired infection, report the risk of retinochoroiditis to vary from 2% in North Eastern Brazil to 25% in Southern Brazil.[4], [9]–[11] No comparable studies have been done in Europe or North America but case series report risks of 0.3% to 1% in adults in the year or two after acquisition of infection.[12]–[14] The fact that retinochoroiditis results in more and larger lesions in South America than in Europe or North America is well accepted by clinicians but differences could reflect delayed presentation due to poor access to health care, referral bias, acquisition of infection from oocysts rather than tissue cysts, or exposure to a higher parasite load or to infection earlier in childhood in Brazil.[15] No published studies have directly compared ocular sequelae of toxoplasmic infection in Brazil with Europe or North America.

Differences in the ocular sequelae of toxoplasmosis have been attributed to recent findings that distinctly different populations of *Toxoplasma gondii* exist in South America compared with Europe and North America.[16],[17] Strains in Brazil appear to be more virulent, and have been identified in several patients with severe ocular disease.[18],[19] In-vitro studies suggest that strain virulence affects triggering of the immune response, tissue penetration and the ability to encyst. As treatment is effective only during the tachyzoite phase, prior to encystment, this raises the possibility that the response to anti-toxoplasma treatment may differ between strains.[20],[21] Possible clinical and policy implications of these findings could be the development of targeted treatment and preventive strategies depending on the prevailing parasite genotype.

Our aim was to inform clinical practice and policy, by quantifying differences in ocular sequelae after toxoplasmic infection in Brazil compared with Europe. We directly compared cohorts of children with congenital toxoplasmosis identified by neonatal or prenatal screening. This approach minimized differences in the route and timing of infection as all fetuses were infected by transplacental transmission of tachyzoites. Health care access was also similar as screening was applied to all births, and we prospectively followed up children using the same protocol in both continents.

## Methods

### Study population

We prospectively recruited and followed up a cohort of live-born children with congenital toxoplasmosis identified between 1996 and 2003 by neonatal or delivery screening in 2 Brazilian centers, by neonatal screening in 3 European centers (Sweden, Denmark, and Poland), and by prenatal screening in 10 European centers (7 in France, 2 in Italy, 1 in Austria). We also included children identified in a prospective national Danish neonatal screening study between 1992 and 1996.[22] Details of screening methods and treatment in the European centers have been reported elsewhere.[22]–[25] In all centers, we excluded any mother or infant who was not first identified by universal screening and who could have been referred for testing because of symptomatology. The diagnosis of congenital toxoplasmosis was based on persistence of specific IgG antibodies after 11.5 months of age.[23],[24]

In Brazil, neonatal screening was based on testing of the neonatal Guthrie card blood spot using an IgM immunoassay (VIDAS, Biomerieux) in Campos Dos Goytacazes, and a commercial capture IgM fluorometric enzyme immunoassay (Labsystems) in Porto Alegre. For screening of mothers at delivery, an IgM immunoassay (VIDAS, BioMérieux) was used.[26],[27] In Campos Dos Goytacazes, neonatal screening was offered to approximately 25% of the total births (about 9,000 births per year) who delivered on two days each week at public sector hospitals within the area. Study infants were recruited between 1999 and 2001. In Porto Alegre, all patients were enrolled who had congenital toxoplasmosis identified between 1998 and 2003 by routine neonatal (*n* = 17) or delivery (*n* = 5) screening and who started follow-up in the Congenital Infections Clinic of Sao Lucas Hospital before 2 months of life (3 patients followed up elsewhere were excluded). Twelve of these 22 children were followed up in the public sector and 10 in the private sector. Postnatal treatment was prescribed for 12 months. Pyrimethamine (1 mg/kg/day) and sulphadiazine (100 mg/kg/day) given for the first six months were changed for the subsequent six months to alternating spiramycin and pyrimethamine-sulphonamide in Campos Dos Goytacazes and to a lower dose of pyrimethamine (1 mg/kg/3 times per week) with sulphonamide (100 mg/kg/day) in Porto Alegre.

### Ophthalmic assessments

We used a standard questionnaire to record findings at routine ophthalmoscopic examinations before 4 months, at 12 months of age, and annually thereafter for all children in the cohort. Clinicians were asked to dilate the pupil and use indirect ophthalmoscopy. They used a standard proforma to record whether the retina was adequately visualized, describe the site of lesions using a diagram and text, and estimate lesion size in multiples of the optic disc diameter. We defined a recurrence as a new lesion that was detected for the first time more than one week after a previous adequate visualization of the retina. Analyses of lesion size were based on the size of the largest lesion at each new occurrence. Multiple lesions were based on the total number of separate lesions detectable at the last examination. We assumed that 2 included children (in Brazil and Europe) had retinochoroiditis although microphthalmia and severe vitreal opacities prevented adequate examination. One of us (MRS) categorized each eye according to whether visual impairment (<6/12 Snellen) was likely or not based on the last retinal diagram. MRS was blinded to visual acuity results.

### Analysis

The incidence of first or recurrent lesions was calculated from the number of new detections and the child years of follow up using a generalized linear model with a Poisson distribution with a log link function and the 95% confidence interval was derived.

Kaplan Meier curves were derived to describe the time to first detection of the first lesion after birth. If a child had no lesions, censoring occurred at the date of the last ophthalmic examination. The probability of lesions by 4 years of age was derived from the product-limit estimates of the survivor function. We used Cox proportional hazards regression to compare differences in the age at detection of the first retinochoroidal lesion in Brazil with European screening centers. As no significant difference was detected between the time to first lesion in European prenatal and neonatal screening centers we compared the size of lesions, risk of recurrence and multiplicity of lesions in the combined European cohort with children in Brazil. The mean size of the largest lesion at each new lesion occurrence was compared using a hierarchical linear model to take into account multiple occurrences in the same child. Results of autoregressive and compound symmetric models were examined to identify the best model (i.e. that with the lowest AIC statistic).[28] Using the dataset combining all children, we had sufficient power to compare the timing of the first and recurrent lesions using methods for multiple failure time data. We used the PWP total time model with common effects[29],[30] which assumes that a subject cannot be at risk for a k^th^ eye lesion unless the (k-1)^th^ lesion has occurred. We chose this model for two reasons. First, the possibility that the infection process or immune response that affects time to initial eye lesion may also impact on times to subsequent lesions, and each period evaluated might present different baseline risks. Second, results were consistent with other approaches, but the PWP model yielded the most conservative hazards ratio.

### Ethics review

Research ethics approval was required for five cohorts where neonatal screening and follow up of positive results was not established routine practice: the two Brazilian centers, patients screened in Denmark between 1992 and 1995, and in Sweden and Poland. In Brazil, screening and follow up was approved by the Ethical Review Board of the Pontificia Universidade Católica do Rio Grande do Sul, Porto Alegre, Brazil, the Ethical Review Board of Fundação Oswaldo Cruz (FIOCRUZ) in Rio de Janeiro, Brazil. Mothers were given information during pregnancy about the screening study and verbal consent was obtained for testing at the time of sampling. Refusal to participate in screen testing was recorded by removing the card used for toxoplasma testing. Separate written consent was obtained from parents of screen positive children to participate in the follow up study. None refused. In the Danish study from 1992–1995, in Sweden and in Poland, mothers were given information during pregnancy or at delivery. At the time of blood sampling, verbal consent was obtained to screen testing and to follow up if found to be positive. Refusal to participate was recorded in writing on the filter paper card. This approach of using oral consent to testing and follow up was approved by the Scientific Ethics Committee of Copenhagen (V100.1689/90 and L 02033), the Ethical Committee at Huddinge Hospital, Stockholm (No. 96-089), and the Scientific Ethical Committee of the Karol Marcinkowski University of Medical Sciences in Poznań.

In all other cohorts (in France, Italy, Austria and Denmark from 1996 onwards) screening and follow up of positive test results was part of universal, routinely provided care. Research ethics approval was not required at the time of the study in any of these centers because all three of the following criteria were met: the study did not involve any modification of routine practice and had no impact on patient care; data collected for the study were confined to information held in routine records of screen test results and follow up examinations; and data were anonymized prior to collation for the study. We specifically requested that the Research Ethics Committee of Great Ormond Street Hospital scrutinize the study and provide written confirmation that research ethics review was not required in the UK before the start of prospective data collection in 1996.

## Results

### Study population

The study compared 30 infected children identified by neonatal screening in Brazil with 71 children identified by neonatal screening (29 in Poland and 42 in Scandinavia) and 210 by prenatal screening in Europe (171 in France, 15 in Italy and 24 in Austria). All had at least one ophthalmoscopic exam and reports of inadequate visualization of the retina were rare. No screen positive children were excluded in Brazil but in Europe, 5 children were excluded due to possible referral bias and a further 3 because they had no ophthalmoscopic exam. The shortest interval between a negative examination and a new lesion occurrence was 22 days. None of the mothers of children in the neonatal centers were treated during pregnancy, whereas 178/210 (85%) in the prenatal centers were treated. All except 3 children (all in Europe) were treated postnatally, mostly for 1 year (further details reported elsewhere).[22],[23],[27] The median age at the start of postnatal treatment was 3 days (IQR: 0, 15) in prenatal centers, 27 days (IQR: 23, 34) in European neonatal centers, and 47 days (IQR 25, 82) in Brazilian centers.

### Occurrence of retinochoroiditis

**Figure 1:**
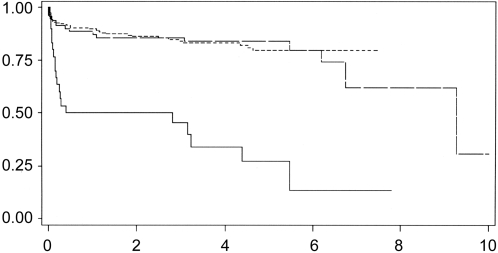
Survival analyses showing proportion of children without retinochoroiditis according to age in years when first eye lesion was detected in Brazil (solid line), and European neonatal (long dash) and prenatal centers (short dash).

The survival analyses in Figure 1 show that more children developed retinochoroiditis sooner in Brazil than in Europe. The hazard ratio shows a markedly increased risk of early retinochoroidal lesion in Brazil than in Europe (Table 1; p<0.0001). There was no evidence that the time to first lesion differed between European neonatal and prenatal screening centers (p = 0.9601; Table 1 and Figure 1). The hazard ratio for time to first retinochoroidal lesion in neonatal centers in Brazil compared with neonatal centers in Europe was 2.37 (95%CI: 1.62, 3.47).

**Table 1:** Occurrence of retinochoroiditis in children with congenital toxoplasmosis in Brazil compared with Europe

	Europe	Europe	Europe	Brazil
	Neonatal	Prenatal	TOTAL	Neonatal
	N = 71	N = 210	N = 281	N = 30*
Period of recruitment	1992–2000	1996–1999		1999–2002
**Examinations**				
Median age (years) at last ophthalmoscopy (range)	4.2 (0.08, 11.46)	4.0 (0.01, 7.50)	4.08 (0.01, 11.46)	3.65 (0.38, 8.47)
Median ophthalmology exams (range)	6 (1, 18)	4 (1, 13)	4 (1, 18)	4 (1, 9)
Indirect ophthalmoscopy/all examinations (%)	251/351 (71)	468/984 (48)	719/1335 (54)	127/135 (94)
**Frequency of retinochoroidal lesions**				
Incidence lesion(s) detection/child year at risk (95%CI)	0.083 (0.056, 0.118)	0.072 (0.055,0.094)	0.076 (0.061, 0.093)	0.334 (0.238, 0.454)
Any retinochoroiditis (%)	16 (23)	34 (16)	50/281 (18)	20 (67)
Any retinochoroiditis in infancy (%)	10 (14)	25 (12)	35/281 (12)	15 (50)
Probability of lesions by 4 years (95%CI)	0.160 (0.073, 0.228)	0.167 (0 112, 0.221)	0.164 (0.118, 0.210)	0.659 (0.465, 0.853)
Hazard ratio for time to first lesion (95%CI)	1.02 (0.54, 1.92) †			5.36 (3.17, 9.08) ‡
**Lesion recurrence (in children with retinochoroiditis) ** §	**15**	**34**	**49**	**19**
Median number of recurrences/affected child (range; IQR)	1 (1 ,3; 1)	1 (1, 3; 1)	1 (1 ,3; 1)	2 (1, 4; 1)
Incidence rate: one or more recurrences/child year at risk	0.208 (0.112, 0.349)	0.206 (0.128, 0.309)	0.207(0.143, 0.287)	0.298 (0.178, 0.463)
Lesion recurrence (%)	7 (44)	14 (41)	21 (42)	12 (60)
Probability of recurrence by 4 years of age (95%CI)	0.196 (0.000, 0.396)	0.343 (0.168, 0.518)	0.290 (0.156, 0.424)	0.428 (0.201, 0.656)
Hazard ratio for time to second lesion from birth (95%CI)	0.47 (0.16, 1.40) †	1	1	2.20 (1.03, 4.67) ‡
Hazard ratio for time to second lesion from first (95%CI)	0.77 (0.29, 2.05) †	1	1	1.82 (0.88, 3.77) ‡
**Multiple lesions (in children with retinochoroiditis)**				
Total number of lesions	35	70	105	66
Median number lesions/affected child (range; IQR)	2 (1,5;3)	2 (1,5;2)	2 (1,5;2)	3 (1,8;3)
Eyes affected by lesions	23 (16)	46 (11)	69 (12)	31 (52)
Eyes with multiple lesions (%)	8 (35)	20 (43)	28 (41)	19 (61)
**Site of lesion**				
Any bilateral lesion (% affected children)	8 (53)	12 (35)	20 (41)	12 (63)
Bilateral posterior pole lesion (% affected children)	1 (7)	6 (18)	7 (14)	8 (42)
Posterior pole lesion (% affected eyes)	9 (39)	30 (65)	39 (57)	25 (81)
**Mean size of lesion** ¶ (range)				
All ages			1.54 (45)	1.62 (29)
<2 years (number)			1.76 (30)	2.32 (17)
2 to 4 years (number)			1.1 (5)	0.71 (6)
>4 years (number)			1.1 (10)	0.52 (6)
**Predicted visual impairment** ||				
Anticipated visual impairment in best eye	1 (7)	4 (12)	5 (10)	11 (37)
Anticipated visual impairment(% affected eyes)	7 (30)	13 (28)	20 (28)	27 (87)

*: Three children from Brazil were included in a previous study by Neto et al.[27]

**†:** Compared with Europe prenatal

**‡:** Compared with Europe total

**§:** Excludes two children (1 Europe prenatal and 1 Brazil) with presumed retinochoroiditis, microphthalmia and vitreal opacities in whom recurrence could not be assessed.

|| Based on ophthalmologist assessment of retinal diagrams. Criteria for impairment is acuity <6/12. Five eyes without diagrams assumed to be unimpaired based on reported site of lesions.

**¶:** Mean size of the largest lesion at each new lesion occurrence measured in disc diameters. Europe datasets combined because of small numbers

IQR = interquartile range

### Recurrence

Retinochoroidal lesion recurred at an earlier age in Brazil than in Europe (p = 0.0406; Table 1). By 4 years of age, the probability of a second lesion among children with a first lesion was 43% in Brazil compared with 29% in Europe (Table 1). The risk of multiple recurrences was also greater in Brazil (hazard ratio for the time from birth to multiple lesions: 3.44; 95%CI: 2.23, 5.32). There was no significant difference in the age at recurrence between European prenatal and neonatal screening centers (p = 0.1740) (Table 1).

### Site of lesions

Children in Brazil were more likely than those in Europe to have retinochoroidal lesions that affected the posterior pole and to have visual impairment predicted by the ophthalmologist assessing the retinal diagrams (p<0.001; Table 1). Relatively more children in Brazil than Europe had multiple lesions (p<0.0001).

### Size of lesions

**Figure 2:**
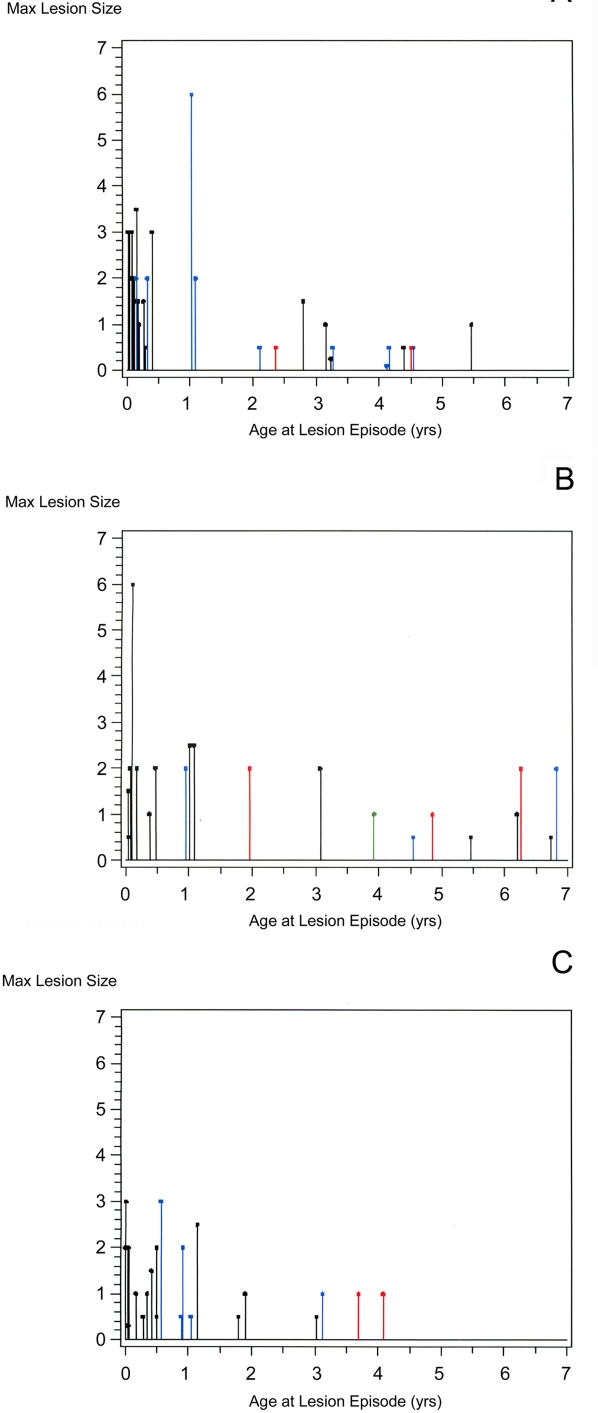
Size measured in disc diameters (y axis) and age (years, x axis) of the first (black), second (blue) and third (red) and fourth (green) newly detected retinochoroidal lesions in (A) Brazil, and (B) European Neonatal Centers and (C) European Prenatal Centers.

The size of the largest lesion was recorded for 62% (74/119) of first or recurrent lesions. Figures 2 A to C depict the size of the largest lesion for each lesion occurrence according to age at detection. The overall mean size was greater in Brazil than in Europe (p<0.0001, Table 1). In both continents, lesion size decreased with the age at detection (p = 0.0007).

## Discussion

Children with congenital toxoplasmosis in Brazil developed retinochoroiditis earlier than children in Europe, had multiple lesions more frequently, and had larger lesions that were more likely to affect the posterior pole and hence to threaten vision. Lesions appeared to diminish in size with age at detection.

This is the first study to compare toxoplasmic retinochoroiditis in concurrent cohorts from Brazil and Europe. Strengths of the study include prospective enrollment and follow up of children in the same way in both continents. A potential source of bias is the fact that Brazilian ophthalmologists have more experience with toxoplasmic retinochoroiditis than their European counterparts and used indirect ophthalmoscopy for virtually all examinations whereas only 54% of examinations were done this way in Europe (73% of European children had at least one indirect examination). Such detection bias is unlikely to explain the very large differences between Brazil and Europe, which persisted for posterior pole lesions, which all ophthalmologists should be able to detect regardless of ophthalmoscopy method.

There are three possible confounding factors that could have contributed to the differences in the risk of retinochoroiditis between Brazil and Europe. Firstly, two-thirds of children in the European cohort received prenatal treatment. We have reported elsewhere that prenatal treatment did not statistically significantly reduce the risk of retinochoroiditis, and similar findings have been reported by others.[23], [31]–[33] Moreover, the differences between Brazil and Europe persisted when we compared neonatal screening centers in Brazil and Europe. The fact that postnatal treatment was started later in Brazil than in European neonatal screening centers would not affect the higher prevalence of lesions at birth in Brazil. Secondly, the mothers of children in Brazil would have seroconverted on average later in pregnancy than those identified by prenatal screening in Europe because IgM screening favors detection of children infected in the latter half of pregnancy.[34] Given the weak association between early gestational age at maternal seroconversion and the risk of retinochoroiditis, our results would be biased in favor of underestimating differences in the risk of retinochoroiditis in Brazil compared with Europe.[23],[31],[32] Thirdly, termination of fetuses with ocular disease in the prenatal centers could have reduced the risk of ocular disease in Europe. This seems unlikely as the number of pregnancy terminations for toxoplasmosis in European prenatal screening centers was very low: 17 out of 1327 women had terminations for toxoplasmosis, of which 9 were infected and very few (n = 6) had evidence of clinical manifestations at autopsy.[35]

Differences between cohorts in calendar time and ethnicity are unlikely to account for the large differences observed between Europe and Brazil. Previous analyses based on European data have shown weak effects of calendar time, with a decreased risk of lesions in later years. This source of bias would lead to an underestimate of the difference between the two continents. Ethnic variation is unlikely to explain the very large differences observed as epidemiological studies have shown that the prevalence of toxoplasmic infection and ocular disease in populations is associated with country of birth and region of early childhood, rather than ethnicity.[36],[37]

Differences between Brazil and Europe are unlikely to be due to anomalies in the regions included in the cohort. Within Europe, increasing latitude has been weakly associated with a diminished risk of clinical manifestations, but the European cohort was representative of all latitudes across western Europe with children recruited from southern, central and northern Europe. The Brazilian cohort was representative of the mix of public and private provision of health care in Brazil where 20% of the population have private health care but our findings differed from a previous cohort study of children with congenital toxoplasmosis in Brazil, which found that the risk of clinical manifestations (intracranial and/or ocular lesions) in the first year of life (17%; 8/47) was very similar to Europe.[27],[31] This low rate of ocular disease in the study by Neto et al may reflect inadequate follow up, a reduced risk in more affluent socioeconomic groups, or regional variation in the incidence of exposure to toxoplasmosis and risk of ocular disease in Brazil.[4],[6],[38] The study by Neto et al was based on children from all regions of Brazil whose families could afford private health care, whereas the present study was based in two endemic areas and only one-third of the children had private health care. However, we found no evidence that the risk of retinochoroiditis differed according to public (12/20) or private sector patients (8/10). Incidence rates of congenital toxoplasmosis in Porto Alegre are also similar in the private and public sectors (private 4.4/10,000 live births; 95%CI : 1.3–9.6, in 1998–2003; public 6.0/10,000; 95%CI: 2.4–12.5, in 2002; personal communication EG Lago, Porto Alegre).

We suggest that the increased frequency and severity of ocular disease in Brazil compared with Europe is due to exposure to more virulent strains of *T.gondii* in Brazil. Type 1 and atypical strains appear to be associated with more severe ocular disease[18],[19] compared with type II strains, which predominate in Europe and North America.[16], [17], [38]–[41] The increased virulence of strains in Brazil compared with Europe may reflect more frequent sexual reproduction in the life-cycle of *T.gondii* in Brazil and human exposure to recombinant strains due to more frequent acquisition of infection via oocysts in Brazil.[42] Recent in-vitro studies suggest that the more virulent strains have a higher rate of growth, a remarkable ability to migrate across epithelial barriers to penetrate host tissue,[43] and can down regulate interleukin 12 more efficiently than the type II strain, which could impair the child's protective immune response.[20],[21],[44]

The finding that the size of lesions diminished with time since infection has not been reported previously. Cautious interpretation is required as detection bias could explain this finding if small lesions are more readily detected as ophthalmoscopy becomes easier with age. Alternatively, lesions may be walled off more effectively as the host immune response matures or the organism load may diminish as bradyzoite cysts involute. Ophthalmologist predictions for visual impairment should also be regarded with caution. These were used as a surrogate measure as children in Brazil did not have visual acuity measures. However, limited accuracy in ophthalmologist prediction (85% specific and 59% sensitive for visual impairment of <6/18 Snellen),[45] is unlikely to explain the very large differences in predicted visual impairment observed between Brazil and Europe.

Further research is required to determine whether virulence factors are associated with prolongation of the tachyzoite phase, which could create a longer therapeutic window before tissue cyst formation when anti-toxoplasmic treatment might be effective. Prospective cohort studies in Europe have shown no evidence for a protective effect of prenatal treatment on ocular disease and data from the EMSCOT study found no effect of postnatal treatment, although the power to detect an effect was limited.[31]–[33] Clearly postnatal treatment was not highly effective in 13/20 children in the Brazil cohort, who developed 17 new lesions while on treatment for at least two weeks (as did 15/50 children with18 lesions in the European cohort). Randomized controlled trials are needed most urgently in South America to determine the effectiveness of postnatal treatment for congenital toxoplasmosis and hence, whether neonatal screening is worthwhile. Extrapolation of results on treatment effectiveness across continents may not be justified in view of the possibility that pharmacodynamics might differ according to parasite genotype.

### Conclusions

In Brazil, congenital toxoplasmosis resulted in more frequent and more severe ocular disease than in Europe. There is indirect evidence that these differences may be related to the predominance of virulent genotypes of the *T.gondii* parasite in Brazil. Randomized controlled trials are urgently needed in South America to determine treatment efficacy and the clinical effectiveness of neonatal screening.

## Supporting Information

Alternative Language Article S1 Translation of the Article into Portuguese by Eleonor G. Lago
(0.34 MB DOC)
