# Supplementary material for: Ocular Sequelae of Congenital Toxoplasmosis in Brazil Compared with Europe
Source: PLoS Negl Trop Dis. 2008 Aug 13;2(8):e277. doi: 10.1371/journal.pntd.0000277 (PMC2493041; doi:10.1371/journal.pntd.0000277)
Supplement: Alternative Language Article S1 — Translation of the Article into Portuguese by Eleonor G. Lago (0.34 MB DOC) [file pntd.0000277.s001.doc]

Seqüelas oculares de toxoplasmose congênita: comparação entre Brasil e Europa

Autores: Ruth E. Gilbert^1§^, Katherine Freeman^2^, Eleonor G. Lago^3^, Lilian M.G. Bahia-Oliveira^4^, Hooi Kuan Tan^1^, Martine Wallon^5^, Wilma Buffolano^6^, Miles R. Stanford ^7^, Eskild Petersen^8^ pelo The European Multicentre Study on Congenital Toxoplasmosis (EMSCOT)*

* membros relacionados no final do artigo

^1^Centro de Epidemiologia e Bioestatística Pediátrica, Institute of Child Health, Londres, Reino Unido

^2^ Departamento de Epidemiologia e Saúde Populacional, Albert Einstein College of Medicine, Nova Iorque, Estados Unidos.

^3^Infecções Congênitas, Departamento de Pediatria, Pontificia Universidade Católica do Rio Grande do Sul, Porto Alegre, Brasil

^4^Laboratório de Biologia do Reconhecer, Centro de Biociências Biotecnológicas, Universidade Estadual do Norte Fluminense, Campos dos Goytacazes, Brasil

^5^Laboratório de Parasitologia Exótica, Hôpital de la Croix Rousse, Lyon, França

^6^Unidade de Infecções Perinatais, Departamento de Pediatria, Universidade Napoli Federico II, Nápolis, Itália

^7^ Departamento de Oftalmologia Acadêmica, Kings College London, Londres, Reino Unido

^8^Departamento de Doenças Infecciosas, Aarhus University Hospital, Aarhus, Dinamarca

^§^Autor correspondente

Titulo resumido: Toxoplasmose ocular no Brasil *versus* Europa

Contagem de palavras - 3260

**RESUMO**

**Introdução**

Embora ainda não tenha sido feita uma comparação direta, a retinocoroidite toxoplásmica parece ser mais severa no Brasil, onde representa a principal causa de cegueira, do que na Europa. Progressivamente, vêm sendo acumuladas evidências de que na América do Sul predominam os genotipos mais virulentos de *Toxoplasma gondii*.

## Métodos

Comparamos coortes prospectivas de crianças com toxoplasmose congênita, identificada por triagem neonatal universal no Brasil e triagem pré-natal ou neonatal na Europa, entre 1992 e 2003, utilizando o mesmo protocolo em ambos os continentes.

## Resultados

Foram estudadas 331 crianças com toxoplasmose congênita: 30 no Brasil e 281 na Europa, onde 71 foram identificadas por triagem neonatal. O seguimento médio foi de 4,1 anos na Europa e 3,7 anos no Brasil. Mais crianças, relativamente, tiveram retinocoroidite durante o primeiro ano no Brasil do que na Europa (15/30; 50% vs 29/281; 10%) e o risco de lesões aos 4 anos de idade foi muito mais alto: razão de chances de 5,36 (IC 95%: 3,17-9,08) para Brasil versus Europa. As crianças brasileiras tiveram lesões maiores, mais freqüentemente múltiplas e afetando o polo posterior (p<0,0001). Foi predita deficiência visual (Snellen <6/12) para a maioria dos olhos afetados no Brasil, (87%, 27/31), mas não na Europa (29%; 20/69, p<0,0001). O tamanho das lesões novas decresceu com a idade (p= 0,0007).

## Conclusões

O *Toxoplasma gondii* causa doença ocular mais grave nas crianças infectadas de forma congênita no Brasil, comparando com a Europa. As diferenças marcantes na freqüência, tamanho e quantidade de lesões de retinocoroidite podem ser devidas à infecção com os genotipos mais virulentos do parasita que predominam no Brasil e são mais raramente encontrados na Europa.

**SUMÁRIO**

O *Toxoplasma gondii* é encontrado em todo o mundo e é a infecção parasitária mais comum nos seres humanos. A infecção pode causar lesões inflamatórias no polo posterior dos olhos, que em alguns casos afetam a visão. Essas complicações parecem ser mais comuns e mais graves quando as pessoas adquirem a infecção no Brasil do que na Europa ou na América do Norte, mas ainda não havia sido feita uma comparação direta entre pacientes identificados e acompanhados com a mesma metodologia. Nesta comunicação, comparamos crianças com toxoplasmose congênita diagnosticada ao nascimento por triagem universal na Europa e no Brasil e acompanhadas até os 4 anos de idade. Encontramos que as crianças brasileiras tiveram um risco 5 vezes maior do que as européias de desenvolver lesões oculares e suas lesões foram maiores, mais numerosas e mais freqüentemente afetando a área da retina responsável pela visão central. Dois terços das crianças brasileiras com toxoplasmose congênita tinham lesões oculares aos 4 anos de idade, comparadas com uma em cada 6 européias. Essas nítidas diferenças são possivelmente devidas ao predomínio de genotipos mais virulentos do parasita no Brasil, os quais são raramente encontrados na Europa.

**INTRODUÇÃO**

A retinocoroidite toxoplásmica parece ser mais comum e mais grave no Brasil do que na Europa ou na América do Norte. Representa a principal causa de cegueira no Brasil [1] mas não na Europa ou na América do Norte. [2,3] Essas diferenças não são suficientemente explicadas pelos altos índices de infecção pós-natal no Brasil, já que índices similares têm sido observados na França e na Europa oriental. [4–8]

Estudos populacionais em adolescentes e adultos infectados, na maioria de forma adquirida, mostram um risco de retinocoroidite que varia entre 2% na região Sudeste e 25% na região Sul do Brasil. [4,9–11] Não foram realizados estudos similares na Europa e na América do Norte, mas algumas séries de casos referem riscos de 0,3% a 1% nos adultos, dentro de um ou dois anos após adquirir a infecção. [12–14] O fato de que a retinocoroidite resulta em lesões maiores e mais numerosas na América do sul do que na Europa e na América do Norte é bem aceito pelos clínicos, mas as diferenças poderiam refletir a ocorrência, no Brasil, das seguintes situações: diagnóstico mais tardio devido ao deficiente acesso ao sistema de saúde; viés de referência; aquisição da infecção por oocistos ao invés de cistos teciduais; exposição a um inóculo parasitário maior; e/ou infecção mais precoce, durante a infância. [15] Nenhum estudo publicado comparou diretamente as seqüelas oculares da infecção toxoplásmica entre Brasil e Europa ou América do Norte.

Diferenças entre as seqüelas oculares da toxoplasmose vêm sendo atribuídas a recentes descobertas, na América do Sul, da existência de populações de *Toxoplasma gondii* diferentes das encontradas na Europa e na América do Norte. [16,17] As cepas no Brasil parecem ser mais virulentas e foram identificadas em pacientes com doença ocular severa. [18,19] Estudos *in vitro* sugerem que a virulência da cepa afeta o estímulo à resposta imune, a penetração tecidual e a capacidade de encistamento. Como o tratamento somente é eficaz sobre o estágio de taquizoíto, antes da formação do cisto tecidual, o fato levanta a possibilidade de que a resposta ao tratamento possa ser diferente entre as diferentes cepas. [20,21] As possíveis implicações desses achados, tanto clínicas quanto em relação a políticas de saúde, podem ser o desenvolvimento de tratamentos específicos e de estratégias preventivas conforme o genotipo prevalente do parasita.

Nosso objetivo foi fornecer informações para a prática clínica e para o planejamento de estratégias de controle, quantificando as diferenças nas seqüelas oculares conseqüentes à infecção toxoplásmica no Brasil, em comparação com a Europa. Comparamos diretamente coortes de crianças com toxoplasmose congênita identificadas por triagem neonatal ou pré-natal. Esta abordagem minimizou as diferenças na via e no tempo de infecção, uma vez que todos os fetos foram infectados por transmissão transplacentária de taquizoítos. O acesso aos cuidados de saúde foram também similares, uma vez que a triagem foi aplicada a todos os nascimentos em determinado período, e as crianças foram acompanhadas com o mesmo protocolo em ambos os continentes.

**MÉTODOS**

*População de estudo*

Recrutamos e acompanhamos uma coorte de crianças com toxoplasmose congênita, identificadas entre 1996 e 2003, através de triagem neonatal ou no momento do parto em 2 centros brasileiros (Campos dos Goytacazes/RJ e Porto Alegre/RS), através de triagem neonatal em 3 centros europeus (Suécia, Dinamarca e Polônia) e através de triagem pré-natal em 10 centros europeus (7 na França, 2 na Itália e 1 na Áustria). Incluímos também crianças identificadas em um estudo nacional prospectivo de triagem neonatal realizado na Dinamarca entre 1992 e 1996. [22] Detalhes dos métodos de triagem e do tratamento nos centros europeus foram relatados em outra publicação. [22–25] Em todos os centros excluímos qualquer paciente que não tenha sido identificado por triagem universal, em que a mãe ou a criança pudessem ter sido encaminhadas para avaliação devido a sintomas sugestivos de toxoplasmose. O diagnóstico de toxoplasmose congênita baseou-se na persistência de anticorpos IgG anti-*Toxoplasma gondii* após 11,5 meses de idade. [23,24]

No Brasil, a triagem neonatal consistiu na testagem de sangue capilar em papel filtro, utilizando um enzimaimunoensaio fluorométrico de captura para IgM (LabSystems, Helsinque, Finlândia). Para os testes confirmatórios e para a triagem das mães no momento do parto foi utilizado o teste enzimático com substrato fluorescente (VIDAS, bioMérieux, Lyon, França). [26,27]

Em Campos dos Goytacazes a triagem neonatal foi oferecida a aproximadamente 25% do total de nascimentos (cerca de 9.000 nascimentos por ano) ocorridos em dois dias por semana nos hospitais públicos do município. Os pacientes foram recrutados para o estudo entre 1999 e 2001. Em Porto Alegre, foram incluídos todos os pacientes com toxoplasmose congênita identificados entre 1998 e 2003 por triagem neonatal de rotina (n=17) ou triagem materna de rotina no momento do parto (n=5) e que iniciaram o seguimento no Ambulatório de Infecções Congênitas do Hospital São Lucas da PUCRS antes dos 2 meses de idade (foram excluídos 3 pacientes acompanhados em outros serviços). Dos 22 pacientes, 12 foram acompanhados no setor público e 10 no setor privado. O tratamento pós-natal foi administrado por 12 meses e consistiu de pirimetamina (1mg/kg/dia) e sulfadiazina (100mg/kg/dia), mais ácido folínico. Em Campos dos Goytacazes, após os primeiros 6 meses, esse esquema foi alternado com espiramicina. Em Porto Alegre, após os primeiros 6 meses a pirimetamina foi administrada na dose de 1mg/kg em 3 doses semanais.

*Avaliações oftalmológicas*

Utilizamos um questionário padrão para anotar os achados das avaliações oftalmoscópicas de rotina realizadas antes dos 4 meses de idade, aos 12 meses e, após, anualmente, em todas as crianças da coorte. Os clínicos eram solicitados a dilatar a pupila e usar oftalmoscópio indireto. Eles utilizavam um protocolo padrão para anotar se a retina era facilmente visualizável, descrever a localização das lesões textualmente e através de um diagrama e estimar o tamanho das lesões em múltiplos do diâmetro discal. Definimos recorrência quando uma nova lesão era detectada, pela primeira vez, mais de uma semana após uma prévia visualização adequada da retina. As análises que levaram em conta o tamanho das lesões consideraram a maior lesão a cada nova ocorrência. O número de lesões foi baseado no número total de lesões separadas, detectadas no último exame. Consideramos que 2 crianças (uma no Brasil e uma na Europa) tinham retinocoroidite, embora a visualização da retina tenha sido prejudicada por microftalmia e opacidade vítrea intensa. Um de nós (MRS), cegado para resultados de acuidade visual, categorizou cada olho conforme a deficiência visual (<6/12 Snellen) fosse ou não provável, com base no último diagrama da retina.

*Análise*

A incidência da primeira lesão ou das recorrências foi calculada pelo número das novas detecções e dos anos de acompanhamento da criança, utilizando um modelo linear generalizado com distribuição de Poisson e com função logarítmica, sendo calculado o intervalo de confiança de 95%.

Foram geradas curvas de Kaplan-Meier para descrever o tempo transcorrido entre o nascimento e a detecção da primeira lesão de retinocoroidite. Nas crianças que não apresentaram nenhuma lesão, a data da censura correspondeu à da última avaliação oftalmológica. A probabilidade de lesões aos 4 anos de idade foi derivada do estimador do produto limite para a função sobrevivência. Utilizamos a regressão de risco proporcional de Cox para comparar diferenças na idade de detecção da primeira lesão de retinocoroidite entre os centros brasileiros e europeus. Como não foi detectada nenhuma diferença significativa no tempo de ocorrência da primeira lesão entre os centros europeus de triagem pré-natal e os de triagem neonatal, consideramos a coorte européia combinada ao comparar o tamanho, o risco de recorrência e a multiplicidade das lesões entre as crianças da Europa e do Brasil. O tamanho médio da maior lesão a cada ocorrência de nova lesão foi comparado através de um modelo linear hierárquico que levou em consideração múltiplas ocorrências na mesma criança. Os resultados dos modelos auto-regressivo e simétrico composto foram examinados para identificar o melhor modelo (ou seja, de menor valor conforme o Critério de Informação de Akaike). [28] Usando o banco de dados completo com todos os pacientes, houve poder estatístico suficiente para comparar a época de surgimento da primeira lesão ocular e das lesões recorrentes, utilizando métodos para dados de tempos de falha múltiplos. Usamos o modelo de tempo total PWP (Prentice, Williams e Peterson) com efeitos comuns [29,30], que considera que um sujeito não pode estar em risco de uma enésima lesão ocular a não ser que tenha ocorrido a lesão (n-1).

Escolhemos este modelo por duas razões. Primeiro, pela possibilidade de que o processo infeccioso ou a resposta imune, que afetam o tempo de aparecimento da primeira lesão, também tenham impacto sobre o tempo de aparecimento das lesões subseqüentes. Segundo, os resultados foram consistentes com outras abordagens, mas o modelo PWP apresentou a razão de chances mais conservadora.

*Aspectos éticos*

A aprovação dos comitês de ética em pesquisa foi necessária para cinco coortes provenientes de locais onde a triagem neonatal e o seguimento dos casos positivos não era rotina estabelecida: os pacientes dos dois centros brasileiros, os pacientes triados na Dinamarca entre 1992 e 1995, os da Suécia e os da Polônia. No Brasil, a triagem e o seguimento foram aprovados pelo Comitê de Ética em Pesquisa da Pontifícia Universidade Católica do Rio Grande do Sul, Porto Alegre/RS e pelo Comitê de Ética em Pesquisa da Fundação Oswaldo Cruz (FIOCRUZ), Rio de Janeiro/RJ. As mães foram informadas sobre o estudo durante a gestação, e foi obtido consentimento verbal no momento da coleta de sangue para a triagem. Em caso de recusa em participar do estudo, o cartão seria removido do malote enviado para pesquisa de IgM anti-*Toxoplasma gondii*. Para o acompanhamento das crianças positivas na triagem, os pais eram convidados a assinar um termo de consentimento pós-informação. Não houve nenhuma recusa. No estudo dinamarquês de 1992-1995, assim como na Suécia e na Polônia, as mães eram informadas durante a gestação ou no momento do parto. Por ocasião da coleta de sangue, era obtido consentimento verbal para a triagem e para o seguimento, caso esta fosse positiva. A recusa em participar era anotada no cartão de papel filtro. Essa abordagem de usar o consentimento verbal para a triagem e o seguimento foi aprovada pelo Comitê de Ética em Ciência de Copenhague (V100.1689/90 e L 02033), pelo Comitê de Ética do Huddinge Hospital, Estocolmo (No. 96-089) e pelo Comitê Ético Científico da Universidade de Ciências Médicas Karol Marcinkowski de Poznań, Polônia.

Em todas as outras coortes (na França, na Itália, na Áustria e na Dinamarca de 1996 em diante) a triagem e o seguimento dos casos com testes positivos faziam parte dos cuidados de rotina universais. A aprovação por comitês de ética em pesquisa não foi exigida para o estudo em nenhum desses centros, porque todos os três critérios seguintes foram satisfeitos: o estudo não envolveu qualquer modificação na prática de rotina e não teve impacto nos cuidados aos pacientes; a coleta de dados para o estudo foi restrita às informações contidas nas anotações de rotina dos testes de triagem e dos exames de acompanhamento; e os dados foram tornados anônimos antes da anotação para o estudo. Solicitamos especificamente ao Comitê de Ética em Pesquisa do Great Ormond Street Hospital para que analisasse o estudo e fornecesse uma confirmação por escrito de que no Reino Unido não havia sido exigida a revisão por um comitê de ética antes do início da coleta de dados prospectiva em 1996.

**RESULTADOS**

*População de estudo*

O estudo comparou 30 crianças infectadas, identificadas por triagem neonatal no Brasil, com 71 crianças identificadas por triagem neonatal (29 na Polônia e 42 na Escandinávia) e 210 crianças identificadas por triagem pré-natal (171 na França, 15 na Itália e 24 na Áustria). Todas tiveram pelo menos um exame oftalmoscópico, sendo que relatos de visualização inadequada da retina foram raros. No Brasil nenhuma das crianças triadas foi excluída, mas na Europa 5 crianças foram excluídas devido a possível viés de referência e 3 crianças por não terem sido submetidas a nenhum exame oftalmoscópico. O intervalo mais curto entre um exame negativo e a ocorrência de uma lesão nova foi de 22 dias. Nenhuma das mães das crianças dos centros onde a triagem foi neonatal haviam sido submetidas a tratamento, enquanto que 178/210 (85%) das mães nos centros onde a triagem foi pré-natal foram tratadas durante a gestação. Todas as crianças, exceto 3 européias, foram tratadas após o nascimento, a maioria por 1 ano (mais detalhes foram descritos anteriormente). [22,23,27] A idade média no início do tratamento pós-natal, nos centros que faziam triagem pré-natal, foi de 3 dias (amplitude entre quartis: 0, 15); nos centros europeus de triagem neonatal, foi de 27 dias (amplitude entre quartis: 23, 24); e nos centros brasileiros, foi de 47 dias (amplitude entre quartis: 25, 82).

*Ocorrência de retinocoroidite*

As análises de sobrevivência da Figura 1 mostram que, no Brasil, mais crianças desenvolveram retinocoroidite mais precocemente, do que na Europa. A razão de chances mostra um risco acentuadamente maior de lesão precoce no Brasil do que na Europa (Tabela 1; p<0,0001). Não houve nenhuma evidência de que o tempo de aparecimento da primeira lesão tenha sido diferente entre os centros europeus de triagem neonatal e pré-natal (p=0,9601; Tabela 1 e Figura 1). A razão de chances para tempo da primeira lesão nos centros de triagem neonatal do Brasil comparados com os centros de triagem neonatal da Europa foi de 2,37 (IC 95%: 1,62-3,47).

*Recorrência*

Houve recorrência de retinocoroidite em uma idade mais precoce no Brasil do que na Europa

(p= 0,0406; Tabela 1). Aos 4 anos de idade, a probabilidade de uma segunda lesão entre as crianças com uma lesão prévia foi de 43% no Brasil e de 29% na Europa (Tabela 1). O risco de múltiplas recorrências também foi maior no Brasil (razão de chances para o tempo transcorrido entre o nascimento e a ocorrência de múltiplas lesões: 3,44; IC 95% 2,23-5,32). Não houve diferença estatisticamente significativa na idade de recorrência entre os centros europeus de triagem pré-natal e neonatal (p= 0,1740) (Tabela 1).

*Localização e multiplicidade das lesões*

As crianças no Brasil foram mais propensas a desenvolver lesões de retinocoroidite localizadas no polo posterior e a ter deficiência visual predita pelo oftalmologista que analisou os diagramas retinianos (p <0,001; Tabela 1). Uma porcentagem maior de crianças brasileiras tiveram múltiplas lesões em comparação às européias (p<0,0001).

*Tamanho das lesões*

O tamanho da maior lesão foi anotado em 62% (74 /119) dos casos de primeira lesão ou recorrências. As figuras 2a) a 2c) descrevem o tamanho da maior lesão para cada ocorrência, conforme a idade em que foi detectada. O tamanho médio em geral foi maior no Brasil do que na Europa (p<0,0001, Tabela 1). Em ambos os continentes, o tamanho das lesões diminuiu com a idade de detecção (p= 0,0007).

### DISCUSSÃO

Neste estudo comparativo de crianças com toxoplasmose congênita no Brasil e na Europa, verificou-se que as crianças brasileiras desenvolveram retinocoroidite mais cedo do que as européias, tiveram mais freqüentemente múltiplas lesões e tiveram lesões maiores, que mais vezes afetaram o polo posterior e, conseqüentemente, prejudicaram a visão. As lesões diminuíram em tamanho com a idade de detecção.

Este é o primeiro estudo que compara a retinocoroidite toxoplásmica em coortes simultâneas do Brasil e da Europa. São pontos fortes do estudo a inclusão prospectiva e o mesmo protocolo de seguimento das crianças em ambos os continentes. Uma fonte potencial de viés é o fato dos oftalmologistas brasileiros terem mais experiência com toxoplasmose congênita do que os seus colegas europeus e usarem oftalmoscópio indireto em virtualmente todos os exames, enquanto somente 54% dos exames foram feitos dessa forma na Europa (embora 73% da crianças européias tenham sido, pelo menos uma vez, examinadas com oftalmoscópio indireto). É pouco provável que este viés de detecção explique as enormes diferenças entre o Brasil e a Europa, as quais persistem para as lesões de polo posterior, facilmente visualizadas por qualquer oftalmologista, independentemente do método oftalmoscópico.

Há três possíveis fatores de confusão que podem ter contribuído para as diferenças entre o risco de retinocoroidite entre o Brasil e a Europa. Primeiro, dois terços das crianças nas coortes européias receberam tratamento pré-natal. Nós relatamos anteriormente que o tratamento pré-natal não reduz significativamente o risco de retinocoroidite, e achados similares foram descritos por outros autores. [23,31-33] Adicionalmente, as diferenças entre o Brasil e a Europa persistiram quando comparamos somente os centros de triagem neonatal em cada continente. O fato de que o tratamento pós-natal foi iniciado mais tarde no Brasil do que nos centros de triagem neonatal da Europa não afetaria a alta prevalência de lesões ao nascimento no Brasil. Segundo, as mães das crianças no Brasil poderiam ter-se soroconvertido mais precocemente na gestação do que as identificadas por triagem pré-natal na Europa, pois a triagem de IgM favorece a detecção de crianças infectadas na segunda metade da gestação. [34] Dada a fraca associação entre soroconversão materna mais precoce e risco de retinocoroidite, nossos resultados deveriam sofrer viés em favor da subestimação do risco de retinocoroidite no Brasil em comparação com a Europa. [23,31,32] Terceiro, as interrupções da gestação nos casos de fetos com doença ocular nos centros de triagem pré-natal poderiam ter reduzido o risco de lesão ocular na Europa. Isto parece improvável porque o número de interrupções da gestação por toxoplasmose nos centros europeus de triagem pré-natal é muito baixo: 17 entre 1327 mulheres interromperam a gestação por causa de toxoplasmose, entre as quais 9 fetos estavam infectados e somente 6 tinham evidências de manifestações clínicas à autópsia. [35]

É improvável que diferenças no calendário e na etnia das coortes respondam pelas grandes diferenças observadas entre a Europa e o Brasil. Análises prévias baseadas em dados europeus mostraram efeitos temporais fracos, com diminuição do risco de lesões nos últimos anos. Esta fonte de viés teria levado a subestimar as diferenças entre os dois continentes. Variações étnicas dificilmente explicariam as grandes diferenças encontradas, pois estudos epidemiológicos mostraram que a prevalência de infecção toxoplásmica e de doença ocular nas populações associam-se mais com o país de nascimento e com a região onde foi passada a primeira infância do que com a etnia. [36,37]

As diferenças entre o Brasil e a Europa não devem ser devidas a peculiaridades das regiões de onde procediam as coortes. Dentro da Europa, a latitude crescente tem sido fracamente associada com risco reduzido de manifestações clínicas, mas a coorte européia foi representativa de todas as latitudes da Europa Ocidental, com crianças recrutadas do sul, centro e norte do continente. A coorte brasileira foi representativa da mistura entre pacientes atendidos pelos sistemas de saúde público e privado no Brasil, onde 20% da população tem atendimento privado. Entretanto, nossos dados diferem de uma coorte prévia de crianças com toxoplasmose congênita no Brasil, que encontrou um risco de manifestações clínicas (lesões cerebrais e/ou oculares) no primeiro ano de vida (17%; 8/47) bastante similar ao europeu. [27,31] Esse baixo risco de doença ocular no estudo de Neto et al. pode refletir seguimento inadequado, risco reduzido em grupos mais favorecidos economicamente, ou variações regionais na incidência da exposição à toxoplasmose no Brasil. [4,6,38] O estudo de Neto et al. foi baseado em crianças de todas as regiões do Brasil cujas famílias podem pagar um atendimento de saúde privado, enquanto que o presente estudo foi baseado em duas áreas endêmicas e somente um terço das crianças tinham atendimento privado. Entretanto, não encontramos evidências de que o risco de retinocoroidite tenha sido diferente entre as crianças com atendimento público (12/20) ou privado (8/10). A incidência de toxoplasmose congênita em Porto Alegre também é similar entre os setores privado e público (privado 4,4/10.000 nascidos vivos; IC 95%: 1,3-9,6 em 1998-2003; público 6,0/10.000; IC 95%: 2,4-12,5, em 2002; comunicação pessoal EG Lago, Porto Alegre).

Nossa hipótese é de que a maior freqüência e a acentuada gravidade da doença ocular no Brasil em comparação com a Europa resultam da exposição a cepas mais virulentas de *T.gondii* no Brasil. As cepas do Tipo I e as atípicas parecem associar-se com doença ocular mais severa [18,19] do que as cepas do Tipo II, que predominam na Europa e na América do Norte. [16,17,38-41] A virulência aumentada das cepas predominantes no Brasil em comparação com as que predominam na Europa podem refletir a reprodução sexual mais freqüente dentro do ciclo vital do *T.gondii* no Brasil, assim como maior exposição humana a cepas recombinantes, devida ao fato de, no Brasil, a aquisição da infecção dar-se mais freqüentemente através de oocistos. [42] Estudos recentes *in vitro* sugerem que as cepas mais virulentas apresentam uma velocidade de multiplicação mais rápida, uma marcante capacidade de migrar através das barreiras epiteliais e penetrar nos tecidos do hospedeiro, [43] e podem regular a interleucina 12 mais eficientemente do que as cepas do Tipo II, o que poderia prejudicar a resposta imune protetora da criança. [20,21,44]

O achado de diminuição no tamanho das lesões com o tempo transcorrido desde o momento da infecção não havia sido relatado anteriormente. É preciso ter cautela na interpretação desse achado, pois um viés de detecção poderia explicá-lo, uma vez que as lesões pequenas tornam-se mais prontamente detectáveis à medida em que a oftalmoscopia vai ficando mais fácil com o crescimento da criança. Alternativamente, as lesões podem ser mais efetivamente limitadas conforme vai amadurecendo a resposta imune do hospedeiro, ou a carga parasitária pode diminuir com a involução dos cistos teciduais. As predições dos oftalmologistas quanto ao grau de deficiência visual também devem ser olhadas com cautela. Elas foram utilizadas como medida substituta, uma vez que a maioria das crianças brasileiras não tinha avaliação da acuidade visual. Entretanto, a acurácia limitada da predição dos oftalmologistas (especificidade de 85% e sensibilidade de 59% para deficiência visual de <6/18 pela classificação de Snellen), [45] dificilmente explicaria diferenças tão grandes, entre o Brasil e a Europa, no grau de deficiência visual predito.

São necessárias mais pesquisas para determinar que fatores de virulência estão associados com o prolongamento da fase de taquizoíto, que podem criar uma janela terapêutica mais longa antes da formação dos cistos teciduais, enquanto o tratamento anti-toxoplásmico ainda pode ser efetivo. Estudos de coorte prospectivos na Europa não encontraram evidência de efeito protetor do tratamento pré-natal na doença ocular, e dados do estudo EMSCOT não mostraram efeito do tratamento pós-natal, embora o poder estatístico para detectar tal efeito tenha sido limitado. [31-33] Claramente, o tratamento pós-natal não foi altamente efetivo em 13/20 crianças da coorte brasileira, que desenvolveram 17 novas lesões quando já em tratamento por pelo menos duas semanas (assim como 15/50 crianças com 18 lesões na coorte européia). São necessários, urgentemente, estudos randomizados na América do Sul, para determinar a efetividade do tratamento pós-natal para toxoplasmose congênita e, conseqüentemente, se a triagem neonatal é vantajosa. A extrapolação dos resultados sobre a efetividade do tratamento de um continente para outro pode não ser válida, tendo em vista a possibilidade de a farmacodinâmica variar conforme o genótipo do parasita.

*Conclusões*

No Brasil, a toxoplasmose congênita resultou em doença ocular mais freqüente e mais grave do que na Europa. Há evidências indiretas de que essas diferenças possam estar relacionadas à predominância de genótipos mais virulentos do parasita *T.gondii* no Brasil. São necessários, urgentemente, ensaios clínicos randomizados na América do Sul, para determinar a eficácia do tratamento e a efetividade clínica da triagem neonatal.

Referências

1. de Carvalho KM, Minguini N, Moreira F, Kara-Jose N (1998) Characteristics of a pediatric low-vision population. J Pediatr Ophthalmol Strabismus 35: 162-165.

2. de Boer J, Wulffraat N, Rothova A (2003) Visual loss in uveitis of childhood. Br J Ophthalmol 87: 879-884.

3. Suttorp-Schulten MS, Rothova A (1996) The possible impact of uveitis in blindness: a literature survey. Br J Ophthalmol 80: 844-848.

4. de Amorim Garcia CA, Orefice F, de Oliveira LC, Gomes AB, Franca M et al. (2004) Socioeconomic conditions as determining factors in the prevalence of systemic and ocular toxoplasmosis in Northeastern Brazil. Ophthalmic Epidemiol 11: 301-317.

5. Bahia-Oliveira LMG, Abreau AMWde, Azevedo-Silveira J, Orefice F (2001) Toxoplasmosis in southeastern Brazil: an alarming situation of highly endemic acquired and congenital infection. Int J Parasitol 32: 133-137.

6. Bahia-Oliveira LM, Jones JL, Azevedo-Silva J, Alves CC, Orefice F et al. (2003) Highly endemic, waterborne toxoplasmosis in north rio de janeiro state, Brazil. Emerging Infect Dis 9: 55-62.

7. Ancelle T, Goulet V, Tirard-Fleury V, Baril L, Mazaubrun C.du et al. (1996) La Toxoplasmose chez la femme enceinte en France en 1995. Resultats d'une enquete nationale perinatale. Bulletin Epidemiologique Hebdomadiare 51: 227-229.

8. Paul M, Petersen E, Szczapa J (2001) Prevalence of congenital Toxoplasma gondii infection among newborns from the Poznan region of Poland: validation of a new combined enzyme immunoassay for Toxoplasma gondii-specific immunoglobulin A and immunoglobulin M antibodies. J Clin Microbiol 39: 1912-1916.

9. Silveira C, Belfort R, Jr., Muccioli C, Abreu MT, Martins MC et al. (2001) A follow-up study of Toxoplasma gondii infection in southern Brazil. Am J Ophthalmol 131: 351-354.

10. Glasner PD, Silveira C, Kruszon Moran D, Martins MC, Burnier Junior M et al. (1992) An unusually high prevalence of ocular toxoplasmosis in southern Brazil. Am J Ophthalmol 114: 136-144.

11. Portela RW, Bethony J, Costa MI, Gazzinelli A, Vitor RW et al. (2004) A multihousehold study reveals a positive correlation between age, severity of ocular toxoplasmosis, and levels of glycoinositolphospholipid-specific immunoglobulin A. J Infect Dis 190: 175-183.

12. Burnett AJ, Shortt SG, Isaac Renton J, King A, Werker D et al. (1998) Multiple cases of acquired toxoplasmosis retinitis presenting in an outbreak. Ophthalmology 105: 1032-1037.

13. Gilbert RE, Stanford MR (2000) Is ocular toxoplasmosis caused by prenatal or postnatal infection? Br J Ophthalmol 84: 224-226.

14. Perkins ES (1973) Ocular toxoplasmosis. Br J Ophthalmol 57: 1-17.

15. Holland GN (2004) Ocular toxoplasmosis: a global reassessment. Part II: disease manifestations and management. Am J Ophthalmol 137: 1-17.

16. Ajzenberg D, Banuls AL, Su C, Dumetre A, Demar M et al. (2004) Genetic diversity, clonality and sexuality in Toxoplasma gondii. Int J Parasitol 34: 1185-1196.

17. Lehmann T, Marcet PL, Graham DH, Dahl ER, Dubey JP (2006) Globalization and the population structure of Toxoplasma gondii. Proc Natl Acad Sci U S A 103: 11423-11428.

18. Vallochi AL, Muccioli C, Martins MC, Silveira C, Belfort R, Jr. et al. (2005) The genotype of Toxoplasma gondii strains causing ocular toxoplasmosis in humans in Brazil. Am J Ophthalmol 139: 350-351.

19. Khan A, Jordan C, Muccioli C, Vallochi AL, Rizzo LV et al. (2006) Genetic divergence of Toxoplasma gondii strains associated with ocular toxoplasmosis, Brazil. Emerg Infect Dis 12: 942-949.

20. Fux B, Nawas J, Khan A, Gill DB, Su C et al. (2007) Toxoplasma gondii strains defective in oral transmission are also defective in developmental stage differentiation. Infect Immun 75: 2580-2590.

21. Saeij JP, Coller S, Boyle JP, Jerome ME, White MW et al. (2007) Toxoplasma co-opts host gene expression by injection of a polymorphic kinase homologue. Nature 445: 324-327.

22. Lebech M, Andersen O, Christensen NC, Hertel J, Nielsen HE et al. (1999) Feasibility of neonatal screening for toxoplasma infection in the absence of prenatal treatment. Lancet 353: 1834-1837.

23. Gras L, Wallon M, Pollak A, Cortina-Borja M, Evengard B et al. (2005) Association between prenatal treatment and clinical manifestations of congenital toxoplasmosis in infancy: a cohort study in 13 European centers. Acta Paediatrica Scandinavia 94: 1-12.

24. Gilbert R, Gras L (2003) Effect of timing and type of treatment on the risk of mother to child transmission of Toxoplasma gondii. BJOG 110: 112-120.

25. Paul M, Petersen E, Pawlowski ZS, Szczapa J (2000) Neonatal screening for congenital toxoplasmosis in the Poznan region of Poland by analysis of Toxoplasma gondii-specific IgM antibodies eluted from filter paper blood spots. Pediatr Infect Dis J 19: 30-36.

26. Eaton RB, Petersen E, Seppanen H, Tuuminen T (1996) Multicenter evaluation of a fluorometric enzyme immunocapture assay to detect toxoplasma-specific immunoglobulin M in dried blood filter paper specimens from newborns. J Clin Microbiol 34: 3147-3150.

27. Neto EC, Anele E, Rubim R, Brites A, Schulte J et al. (2000) High prevalence of congenital toxoplasmosis in Brazil estimated in a 3-year prospective neonatal screening study. Int J Epidemiol 29: 941-947.

28. Sullivan LM, DKA, Losina E (1999) An Introduction to Hierarchical Linear Modelling. Stat Med 18: 855-888.

29. Littell RC, Milliken GA, Stroup WW, Wolfinger RD (1996) *SAS System for Mixed Models*. SAS Institute Inc:

30. Therneau TM, Grambsch PM (2000) Multiple Events per Subject. In: Modeling Survival Data: Extending the Cox Model. New York: Springer-Verlag. pp. 169-194.

31. The SYROCOT Study Group, Thiebaut R, Leproust S, Chene G, Gilbert RE (2007) Effectiveness of prenatal treatment for congenital toxoplasmosis: a meta-analysis of individual patients' data. Lancet 369: 115-122.

32. Binquet C, Wallon M, Quantin C, Kodjikian L, Garweg J et al. (2003) Prognostic factors for the long-term development of ocular lesions in 327 children with congenital toxoplasmosis. Epidemiol Infect 131: 1157-1168.

33. Freeman K, Tan HK, Prusa A, Petersen E, Buffolano W et al. (2007) Predictors of retinochoroiditis in children with congenital toxoplasmosis: European, prospective cohort study. Pediatrics (in press):

34. Gilbert RE, Thalib L, Tan HK, Paul M, Wallon M et al. (2007) Screening for congenital toxoplasmosis: accuracy of immunoglobulin M and immunoglobulin A tests after birth. J Med Screen 14: 8-13.

35. Thalib L, Gras L, Romand S, Prusa A, Bessieres MH et al. (2005) Prediction of congenital toxoplasmosis by polymerase chain reaction analysis of amniotic fluid. BJOG 112: 567-574.

36. Gilbert RE, Tookey PA, Cubitt WD, Ades AE, Masters J et al. (1993) Prevalence of toxoplasma IgG among pregnant women in west London according to country of birth and ethnic group [see comments]. BMJ 306: 185.

37. Gilbert RE, Dunn D, Lightman S, Murray PI, Pavesio C et al. (1999) Incidence of symptomatic toxoplasma eye disease: aetiology and public health implications. Epidemiol Infect 123: 283-289.

38. Dubey JP, Sundar N, Gennari SM, Minervino AH, Farias NA et al. (2007) Biologic and genetic comparison of Toxoplasma gondii isolates in free-range chickens from the northern Para state and the southern state Rio Grande do Sul, Brazil revealed highly diverse and distinct parasite populations. Vet Parasitol 143: 182-188.

39. Nowakowska D, Colon I, Remington JS, Grigg M, Golab E et al. (2006) Genotyping of Toxoplasma gondii by multiplex PCR and peptide-based serological testing of samples from infants in Poland diagnosed with congenital toxoplasmosis. J Gynecol Obstet Biol Reprod (Paris) 44: 1382-1389.

40. Dubey JP, Su C, Cortes JA, Sundar N, Gomez-Marin JE et al. (2006) Prevalence of Toxoplasma gondii in cats from Colombia, South America and genetic characterization of T. gondii isolates. Vet Parasitol 141: 42-47.

41. Moura L., Bahia-Oliveira LM, Wada MY, Jones JL, Tuboi SH et al. (2006) Waterborne toxoplasmosis, Brazil, from field to gene. Emerg Infect Dis 12: 326-329.

42. Ferreira AM, Vitor RW, Gazzinelli RT, Melo MN (2006) Genetic analysis of natural recombinant Brazilian Toxoplasma gondii strains by multilocus PCR-RFLP. Infect Genet Evol 6: 22-31.

43. Barragan A, Sibley LD (2002) Transepithelial migration of Toxoplasma gondii is linked to parasite motility and virulence. J Exp Med 195: 1625-1633.

44. Saeij JP, Boyle JP, Coller S, Taylor S, Sibley LD et al. (2006) Polymorphic secreted kinases are key virulence factors in toxoplasmosis. Science 314: 1780-1783.

45. Tan HK, Schmidt D, Stanford MR, Teär-Fahnehjelm K, Ferret N et al. (2007) Risk of visual impairment in children with congenital toxoplasmic retinochoroiditis. Am J Ophthalmol 144: 648-653.

**Agradecimentos**

***Membros do *European Multimember Study on Congenital Toxoplasmosis* (EMSCOT)**

*Comitê de redação:* R Gilbert (investigador principal), K Freeman, EG Lago, L Bahia-Oliveira, W Buffolano, HK Tan, M Stanford, M Wallon, E Petersen

*Centros que contribuíram com dados* (número de pacientes incluídos neste estudo): **EUROPA**: P Thulliez, S Romand (64; Institut de Puericulture, Paris, França), M Wallon, F Peyron (44; Hôpital de la Croix Rousse, Lyon, França), E Petersen, D Remmer Schmidt (40; Statenseruminstitut, Copenhague, Dinamarca), M Paul (29; University Medical Sciences, Poznan, Polônia), A Prusa, M Hayde, A Pollak (24; University Children’s Hospital, Viena, Áustria), M-H Bessieres (22; Hôpital de Rangueil, Toulouse, França), J Franck, H Dumon, P Bastien, E Issert (20; Hôpital de la Timone, Marseille; CHU de Montpellier, França), W Buffolano (11; Universita di Napoli, Nápoles, Itália), C Chemla, (8; Hôpital Maison Blanche, Reims, França), N Ferret, P Marty (8; Hôpital de l’Archet, Nice, França), H Pelloux, H Fricker-Hidalgo, C Bost-Bru (6; Centre Hospitalier Universitaire de Grenoble, França), B Evengard, G Malm (3; Huddinge Hospital, Estocolmo, Suécia), E Semprini, V Savasi (4; Milão, Itália). **BRASIL**: EG Lago, J Melamed, E Neto (22; Porto Alegre, Rio Grande do Sul), L Bahia-Oliveira, AM Wilken de Abreu Oliveira, RG Peixe (8; Universidade Estadual do Norte Fluminense Darcy Ribeiro, Campos dos Goytacazes, Rio de Janeiro).

*Delineamento e coordenação do estudo:* R Gilbert (investigador principal), HK Tan (Institute of Child Health, Londres, Reino Unido)

*Análise estatística:* K Freeman, HK Tan, M Cortina-Borja (Institute of Child Health, Londres, Reino Unido)

Somos gratos a Marilia Sa Carvalho, cujos comentários melhoraram a versão final do manuscrito.

**Tabela 1: Ocorrência de retinocoroidite em crianças com toxoplasmose congênita no Brasil e na Europa**

|  | **Europa** | **Europa** | **Europa** | **Brasil** |
| --- | --- | --- | --- | --- |
|  | **Neonatal** | **Pré-natal** | **TOTAL** | **Neonatal** |
|  | N=71 | N=210 | N=281 | N=30^*^ |
| Período de recrutamento | 1992-2000 | 1996-1999 |  | 1999-2002 |
| **Exames** |  |  |  |  |
| Mediana da idade (anos) na última avaliação oftalmológica (variação) | 4,2 (0,08-11,46) | 4,0 (0,01-7,50) | 4,08 (0,01-11,46) | 3,65 (0,38-8,47) |
| Mediana do número de exames oftalmológicos (variação) | 6 (1-18) | 4 (1-13) | 4 (1-18) | 4 (1-9) |
| Oftalmoscopia indireta/todas as oftalmoscopias (%) | 251/351 (71) | 468/984 (48) | 719/1335 (54) | 127/135 (94) |
| **Freqüência das lesões de retinocoroidite** |  |  |  |  |
| Incidência de detecção/criança ano em risco (IC 95%) | 0,083 (0,056-0,118) | 0,072 (0,055-0,094) | 0,076 (0,061-0,093) | 0,334 (0,238-0,454) |
| Alguma lesão de retinocoroidite (%) | 16 (23) | 34 (16) | 50/281 (18) | 20 (67) |
| Alguma lesão de retinocoroidite no primeiro ano de vida (%) | 10 (14) | 25 (12) | 35/281 (12) | 15 (50) |
| Probabilidade de lesão aos 4 anos (IC 95%) | 0,160 (0,073-0,228) | 0,167 (0,112-0,221) | 0,164 (0,118-0,210) | 0,659 (0,465-0,853) |
| Razão de chances para o tempo da primeira lesão (IC 95%) | 1,02 (0,54-1,92) † |  |  | 5,36 (3,17-9,08) ‡ |
| **Recorrência de lesões (em crianças com retinocoroidite) §** | **15** | **34** | **49** | **19** |
| Mediana do número de recorrências/criança afetada (variação; amplitude entre quartis) | 1 (1-3; 1) | 1 (1-3; 1) | 1 (1-3; 1) | 2 (1-4; 1) |
| Taxa de incidência: uma ou mais recorrências/criança ano em risco (amplitude entre quartis) | 0,208  (0,112-0,349) | 0,206  (0,128-0,309) | 0,207  (0,143-0,287) | 0,298  (0,178-0,463) |
| Recorrência de lesões (%) | 7 (44) | 14 (41) | 21 (42) | 12 (60) |
| Probabilidade de recorrência aos 4 anos de idade (IC 95%) | 0,196 (0,000-0,396) | 0,343 (0,168-0,518) | 0,290 (0,156-0,424) | 0,428 (0,201-0,656) |
| Razão de chances para o tempo da segunda lesão desde o nascimento (IC 95%) | 0,47 (0,16-1,40) † | 1 | 1 | 2,20 (1,03-4,67) ‡ |
| Razão de chances para o tempo da segunda lesão desde a primeira (IC 95%) | 0,77 (0,29-2,05) † | 1 | 1 | 1,82 (0,88-3,77) ‡ |
| **Múltiplas lesões (em crianças com retinocoroidite)** |  |  |  |  |
| Número total de lesões | 35 | 70 | 105 | 66 |
| Mediana do número de lesões/criança afetada (variação; amplitude entre quartis) | 2 (1-5; 3) | 2 (1-5; 2) | 2 (1-5; 2) | 3 (1-8; 3) |
| Olhos afetados por alguma lesão | 23 (16) | 46 (11) | 69 (12) | 31 (52) |
| Olhos com múltiplas lesões (%) | 8 (35) | 20 (43) | 28 (41) | 19 (61) |
| **Localização da lesão** |  |  |  |  |
| Bilaterais (% de crianças afetadas) | 8 (53) | 12 (35) | 20 (41) | 12 (63) |
| Bilaterais no polo posterior (% de crianças afetadas) | 1 (7) | 6 (18) | 7 (14) | 8 (42) |
| Alguma lesão de polo posterior (% de olhos afetados) | 9 (39) | 30 (65) | 39 (57) | 25 (81) |
| **Tamanho médio da maior lesão**¶ |  |  |  |  |
| Todas as idades |  |  | 1,54 (45) | 1,62 (29) |
| <2 anos (número) |  |  | 1,76 (30) | 2,32 (17) |
| 2 a 4 anos (número) |  |  | 1,1 (5) | 0,71 (6) |
| > 4 anos (número) |  |  | 1,1 (10) | 0,52 (6) |
| **Deficiência visual predita||** |  |  |  |  |
| **Deficiência visual predita no olho melhor** | 1 (7) | 4 (12) | 5 (10) | 11 (37) |
| **Deficiência visual predita** (% de ohos afetados) | 7 (30) | 13 (28) | 20 (28) | 27 (87) |
|  |  |  |  |  |

* Três crianças do Brasil foram incluídas em estudo anterior de Neto et al.[27]

† Comparação com os pacientes dos centros europeus de triagem pré-natal

‡ Comparação com todos os pacientes europeus

§ Exclui 2 crianças (1 de um centro de triagem pré-natal da Europa e 1 do Brasil) com retinocoroidite presumível, microftalmia e opacidade vítrea, nas quais a recorrência não pôde ser avaliada.

|| Com base na avaliação dos diagramas retinianos por um oftalmologista. O critério para deficiência visual é <6/12 (Senellen). Cinco olhos sem diagramas foram considerados não afetados com base no relato da localização das lesões.

¶ Tamanho médio da maior lesão a cada nova ocorrência de lesão, medida em diâmetros discais. Dados europeus combinados devido ao N pequeno.

LEGENDAS DAS FIGURAS

**Figura 1**: Análise de sobrevida mostrando a proporção de crianças sem retinocoroidite conforme a idade em anos em que foi detectada a primeira lesão, no Brasil (linha sólida), nos centros neonatais da Europa (tracejado longo) e nos centros pré-natais da Europa (tracejado curto).

**Figura 2**:  Tamanho medido em diâmetros discais (eixo Y) e idade em anos (eixo X) da primeira (preto), segunda (azul), terceira (vermelho) e quarta (verde) detecção de nova lesão de retinocoroidite, nos centros do Brasil (A), de triagem neonatal da Europa (B) e de triagem pré-natal da Europa (C).
